# Human Immunodeficiency Virus Infection in Romania Versus Europe: An Epidemiological and Public Health Perspective, 2024 Update

**DOI:** 10.3390/idr18010009

**Published:** 2026-01-09

**Authors:** Andreea-Iuliana Ciobanu, Sebastian Ionescu, Ana Maria Tudor, Mariana Mărdărescu, Laurențiu-Mihăiță Stratan, Adrian Gabriel Marinescu, Cătălin Tiliscan, Aida-Isabela Adamescu, Oana Ganea, Sorin Ștefan Aramă, Victoria Aramă

**Affiliations:** 1 Faculty of Medicine, “Carol Davila” University of Medicine and Pharmacy, 020021 Bucharest, Romania; andreea-iuliana.niculescu@drd.umfcd.ro (A.-I.C.); sebastian.ionescu@umfcd.ro (S.I.); adrian-gabriel.marinescu@drd.umfcd.ro (A.G.M.); catalin.tiliscan@umfcd.ro (C.T.); aida-isabela.adamescu@drd.umfcd.ro (A.-I.A.); oana-alexandra.ganea@drd.umfcd.ro (O.G.); sorin.arama@umfcd.ro (S.Ș.A.); victoria.arama@umfcd.ro (V.A.); 2 “Maria Skłodowska-Curie” Emergency Clinical Hospital for Children, 75534 Bucharest, Romania; 3 “Prof. Dr. Matei Balș” National Institute for Infectious Diseases, 021105 Bucharest, Romania; mardarescum@yahoo.com

**Keywords:** HIV, Romania, epidemiology

## Abstract

Background/Objectives: This study presents a comprehensive and updated epidemiological and public health assessment of human immunodeficiency virus (HIV) in Romania during 2022–2024, situated within the wider European context. Methods: For this retrospective descriptive study, we analyzed national surveillance data from the National Institute of Infectious Diseases “Prof. Dr. Matei Balș” and European Centre for Disease Prevention and Control (ECDC) reports, between 1985–2024, focusing especially on 2022–2024 period. Key indicators included incidence, mortality, transmission routes, age and gender distribution, and treatment coverage. Comparative analyses were performed between Romania and European Union (EU)/Eastern Europe data. Results: Between 1985 and 2024, Romania registered a cumulative total of 28,793 HIV cases, with 18,768 individuals living with HIV (PLHIV) as of 2024. In that year, 810 new HIV cases were diagnoses, indicating a modest uptick compared with 2022–2023. Heterosexual transmission continued to predominate (59.4%), followed by cases among men who have sex with men (MSM) (30.5%) and intravenous drug users (IDUs) (5.2%). Men represented more than three-quarters of all new infections. Mortality displayed considerable year-to-year variability, increasing from 125 HIV-related deaths in 2023 to 193 in 2024. Despite this, treatment coverage improved steadily, with 16,464 individuals receiving antiretroviral therapy (ART) by the end of 2024. At 2.51 cases per 100,000 population, Romania’s incidence remained below the European average of 3.5 per 100,000. Nonetheless, the proportion of infections attributable to MSM transmission rose sharply—from 3.91% in 2007 to 32% in 2024—bringing Romania’s epidemiological profile increasingly in line with broader trends observed in Eastern Europe. Conclusions: These findings suggest that although Romania maintains a comparatively lower HIV incidence than the European average, the evolving transmission dynamics—most notably the substantial increase in MSM-related cases—signal a shifting epidemiological landscape that warrants strengthened, population-specific prevention measures and continued investment in comprehensive treatment and monitoring frameworks.

## 1. Introduction

Human immunodeficiency virus (HIV) infection remains a major global public health concern, exerting deep social, economic, and political impacts. Since its identification in 1983 as the causative agent of Acquired Immunodeficiency Syndrome (AIDS), HIV has driven extensive research efforts and the establishment of comprehensive international programs. Initiatives such as the World Health Organization’s (WHO) Global Program on HIV/AIDS, launched in 1987, have focused on advancing scientific understanding, strengthening prevention strategies, and improving access to treatment [1].

Although initially the reported HIV prevalence in Central and Eastern Europe was low, gradually increased across the region, largely due to delayed testing capacity and the slow adoption of effective prevention measures. Romania was among the first countries in the region to report HIV cases to WHO, documenting its inaugural case in 1985. A major step toward improving epidemiological surveillance was the enactment of Order 200/1987 by the Ministry of Health, mandating centralized reporting of all HIV/AIDS cases to the “Dr. Victor Babeș” Hospital for Infectious Diseases in Bucharest. Nevertheless, early detection efforts were hindered by limited diagnostic infrastructure and restricted access to testing, which remained largely confined to a few specialized laboratories.

A critical turning point in the Romanian epidemic occurred between 1987 and 1990, when an unusually large number of HIV-infected children were identified, drawing international attention. This outbreak was linked to unsafe medical practices, including the reuse of contaminated syringes and equipment in hospitals and child care institutions. This pattern of transmission contrasted sharply with the predominant Western European routes of sexual transmission and intravenous drug use. In response, WHO and the U.S. Centers for Disease Control and Prevention (CDC) partnered with Romanian authorities to implement rapid diagnostic, preventive, and training interventions to contain further spread.

Throughout the 1990s, Romania achieved substantial improvements in HIV diagnosis and treatment. Testing services expanded to reach at-risk groups, including individuals with tuberculosis and sexually transmitted infections. The rise in mother-to-child transmission after 1994 prompted intensified monitoring of pregnant women and the implementation of targeted prevention strategies. Moreover, mandatory screening of donated blood for HIV and hepatitis B was introduced in 1994, significantly reducing the risk of transfusion-related infections.

In 2000, Romania implemented the National Plan for Universal Access to Treatment and Care, a landmark public health initiative that ensured free access to antiretroviral therapy (ART) for all individuals living with HIV. The adoption of more effective treatment regimens and evidence-based prevention strategies under this plan contributed to increased life expectancy and improved quality of life among patients.

Further consolidation of the national response occurred in 2002 with the establishment of the National Multisectoral HIV/AIDS Commission, which strengthened inter-institutional coordination in prevention, surveillance, and patient management. During this period, epidemiological patterns also shifted, with heterosexual transmission becoming increasingly prominent, particularly among adults aged 19–49 years.

Romania has continued to advance its HIV/AIDS response despite persistent challenges. In 2023, the Romanian Government adopted a new National Strategy for the Surveillance, Control, and Prevention of HIV/AIDS, supported by a budget exceeding 1.2 billion lei (approximately €240 million) for the period 2023–2026. The strategy prioritizes expanded access to testing, the integration of HIV screening into primary care services, reinforcement of health education programs, and sustained access to ART. However, limited testing uptake and enduring stigma surrounding HIV infection remain significant obstacles to equitable healthcare access [2].

Given the evolving epidemiological landscape of HIV in Romania and the broader European region, an updated assessment is critically needed to inform national public health priorities. Despite substantial progress in surveillance, prevention, and access to treatment, significant gaps persist—including uneven testing coverage, shifting transmission patterns, and sustained stigma—that may hinder the effectiveness of current strategies. This study was undertaken to provide a contemporary analysis of HIV trends in Romania during 2022–2024, to contextualize these findings within European dynamics and to assess/evaluate Romania’s progress towards UNAIDS 95-95-95 targets. A descriptive epidemiological analysis was conducted using national surveillance data reported to the ‘Dr. Victor Babeș’ Hospital for Infectious Diseases and publicly available European datasets. Key indicators—including incidence, transmission routes, demographic patterns, mortality, and treatment coverage—were examined to evaluate recent trends and identify emerging public health concerns. This methodological approach enables a comprehensive and comparative understanding of Romania’s current HIV burden and supports evidence-based policy planning.

## 2. Materials and Methods

This retrospective descriptive study examined HIV/AIDS surveillance data from Romania spanning 1985–2024, with particular emphasis on epidemiological trends observed between 2022 and 2024, and compared these patterns with corresponding data from the European Union (EU) and Eastern Europe. National data was obtained from the Department for Monitoring and Evaluation of HIV/AIDS Infection at the ‘Prof. Dr. Matei Balș’ National Institute for Infectious Diseases, while European indicators were extracted from reports published by ECDC, UNAIDS, and WHO. The analysis focused on key indicators, including incidence, prevalence, mortality, age and sex distribution, transmission routes, and coverage.

Inclusion criteria comprised all confirmed HIV or AIDS cases reported through the national surveillance system during the study period, as well as all publicly available European datasets containing comparable epidemiological indicators. For better understanding of Romanian epidemiological particularities, we used for comparisons data from neighboring countries, namely Central and Eastern Europe, as well as western European countries, mostly due to similar medical practices, adhering to European for HIV treatment and prevention guideline and inclusion of WHO reports in European Region.

Descriptive statistical methods were applied using Microsoft Excel (Version 14.0.7268.5000, Microsoft Corporation, Redmond, WA, USA), and findings were summarized through tables and graphical representations.

## 3. Results

Between 1985 and 2024, Romania recorded 28,793 cases of HIV, with 18,881 AIDS cases (at least one AIDS related disease or CD4 levels less than 200 cell/mm^3^). By 2024, 1276 individuals had been removed from the registry and 8749 AIDS related deaths had been documented. Currently, 18,768 people are living with HIV (PLWHA). The number of newly reported HIV/AIDS cases between 1 January 2022 and 31 December 2024, was 2233, indicating ongoing viral transmission within the population. Additionally, 553 deaths were recorded during this period (Table 1). The statistical data presented herein are derived from the annual reports of the Department for Monitoring and Evaluation of HIV/AIDS Infection, part of the “Prof. Dr. Matei Balș” National Institute for Infectious Diseases, Bucharest [2].

In Romania, 810 new HIV new cases were registered between 1 January and 31 December 2024, comprising 475 HIV infections and 335 AIDS cases. Compared to 760 new cases reported in 2023 and 757 in 2022, the incidence has shown a slight increase (Table 2) [3].

In 2024, 18,290 HIV/AIDS cases were registered among adults over 20 years of age. In contrast, the number of cases is significantly lower among children in the age group 0–14 years, where 141 cases were reported, and among adolescents (age group 15–19 years) where 130 cases were reported (Table 3).

The most common route of transmission in 2024 was heterosexual contact (59.38%), followed by MSM contact (30.49%) and the use of contaminated injection equipment by intravenous drug users (UDIs) (5.18%) (Table 4). Vertical transmission increased slightly compared to previous years, although it still remains at low level (1.97%). The route of transmission was unknown in six cases (0.74%).

**Figure 1:**
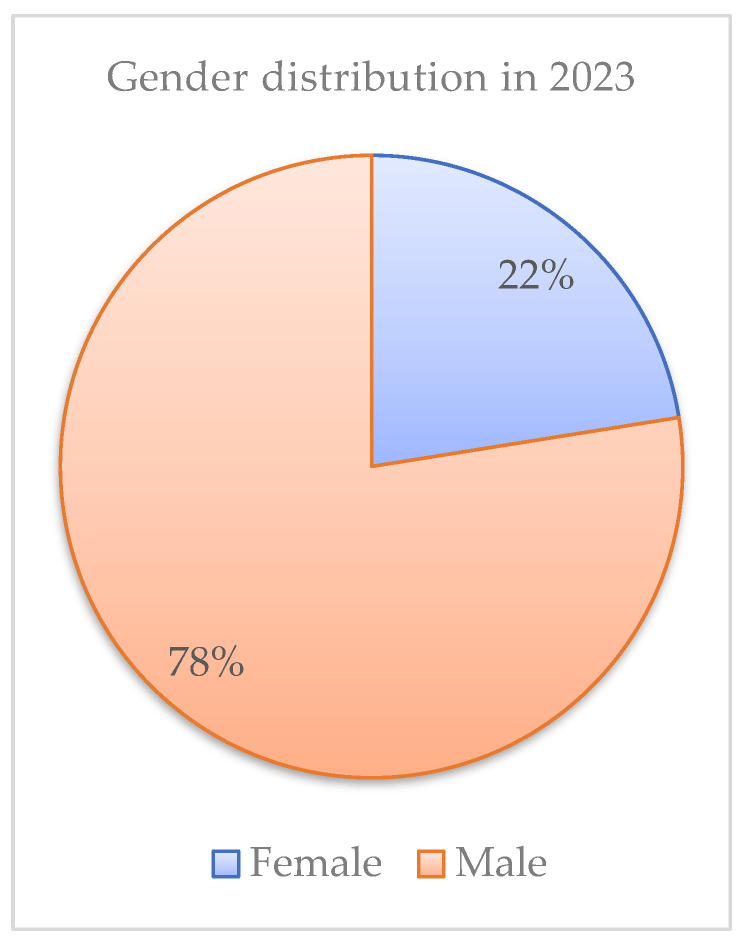
Gender distribution in 2023.

**Figure 2:**
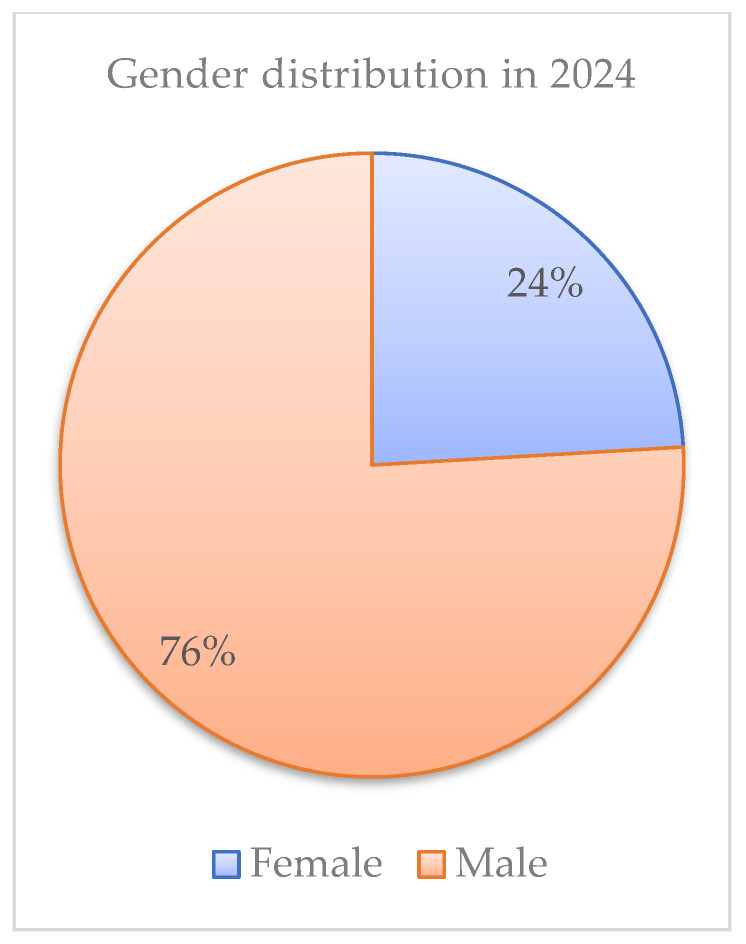
Gender distribution in 2024.

Regarding the gender distribution of new HIV/AIDS cases registered in Romania, in 2023, there was a clear predominance of male patients, representing 77.51% (*n* = 555) of cases, while women represented only 22.49% (*n* = 161) (Figure 1). This trend persisted in 2024, when 75.93% (*n* = 615) of total registered new cases (810) were men and only 24.07% (*n* = 195) were women (Figure 2).

The epidemiologic trends of HIV infection in Romania revealed substantial shifts in the demographic profile of vulnerable populations. The proportion of cases attributed to MSM has risen markedly, from 3.91% in 2007 to 32.67% in 2024, showing a significant change in transmission dynamics. At the same time, it was noted an important decline in transmission among IDUs, from 32.13% in 2012, when the highest number of new cases among iv drug users was recorded, to 5.19% in 2024.

**Figure 3:**
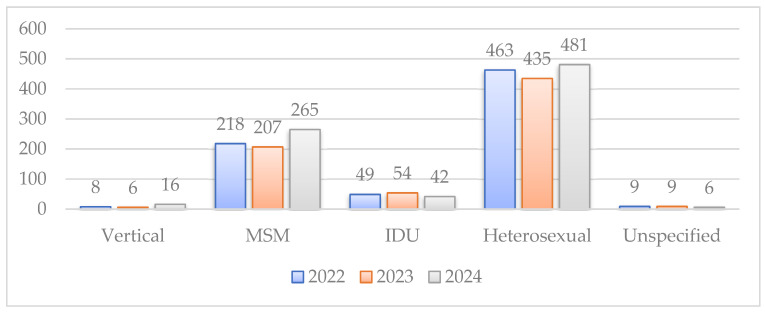
HIV transmission route between 2022–2024.

The mother-to-child transmission (MTCT) rate, however, presents a more complex picture. While demonstrating a slight decrease in 2023, from 0.8% in 2022, a subsequent increase to 1.98% in 2024 (Figure 3).

**Figure 4:**
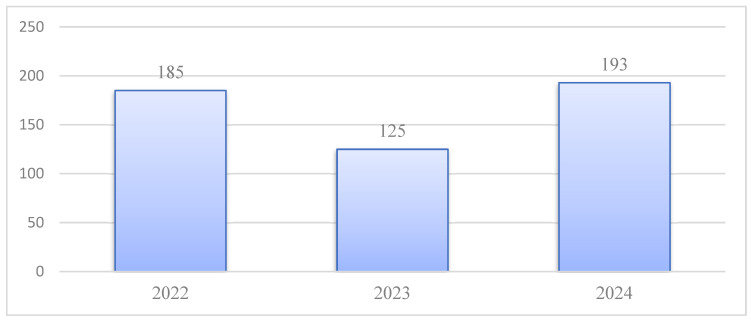
AIDS—related mortality.

Recent data, by the end of December 2024, indicate a relatively stable trend in reported HIV-related mortality over the preceding three years, with 185 deaths in 2022, 125 in 2023, and 193 in 2024 (Figure 4).

The rising incidence of European cases during 2024 suggests an upward trajectory in HIV transmission across the continent, particularly in Eastern Europe. While Romania’s incidence remains below the European average, exhibiting relative stable number of new cases, a slight decline is observed compared to 2023. Although the impact of ART has contributed to a decrease in AIDS-related mortality (deaths) across Europe, a marginal increase in the annual death rate is apparent within Romania (Table 5).

Mortality associated with HIV infection serves as a crucial indicator of the effectiveness of therapeutic interventions, healthcare access, and treatment adherence. The publicly available Romanian data showed an increase in number of HIV patients in care with 22.94%, an increase in number of HIV individuals in ART with 8.49%, but an increase in AIDS related death reported cases with 54% (Table 6).

## 4. Discussion

The epidemiological analysis of HIV in Romania between 2022 and 2024 revealed a complex pattern of both progress and ongoing challenges. Romania experienced an increase in new HIV cases, rising from 716 in 2023 to 810 in 2024, a trend consistent with developments across Europe. The demographic profile of affected individuals mirrors that of neighboring Eastern European countries, with adults comprising the overwhelming majority of cases. In 2024, adults represented 98.55% of all individuals receiving HIV care in Romania, a distribution comparable to Bulgaria (over 99%), Ukraine (95–98%), and Poland (over 97%) [2,3,4,5,6,7]. In contrast, countries with long-established sexual health education and widespread access to pre-exposure prophylaxis (PrEP)—such as Sweden, Norway, and Luxembourg—have seen sustained declines in HIV infections among young people, underscoring the effectiveness of proactive prevention programs [8,9,10].

Transmission patterns during this period highlighted significant shifts that demand targeted intervention. Although heterosexual contact continues to be the predominant route of infection in Romania, accounting for 59.41% of new cases in 2024, the proportion of cases among MSM has risen steadily. Men who have sex with men accounted for approximately 32.67% of new diagnoses in 2024, closely aligning with proportions reported in Poland (32%), Lithuania (30%), and Bulgaria (30%) [1,4,5,7,11,12]. This peak in MSM cases in Romania could be explained by numerous factors, one of them being the fact that in recent years, the stigma toward this population group diminished in our country and the patients were more open to reveal their sexual orientation. This upward trend parallels developments across Central and Eastern Europe and underscores the need for dedicated, population-specific prevention efforts. This necessitates broader, non-discriminatory access to preventative services, notably PrEP. Successful models—such as the United Kingdom’s targeted prevention and community-centered testing initiatives—demonstrate the effectiveness of tailored interventions in reducing incidence among MSM [13].

Transmission among IDUs remains comparatively low in Romania (under 6%), due to efforts to implement risk reduction programs, such as syringe exchange and opioid substitution treatment, especially after highest number of cases among this vulnerable population group in 2012. International experience underscores the importance of maintaining robust harm reduction services, access to NEPs OST is therefore crucial. The 2023 ECDC report and the 2022 UNAIDS global update confirm that comprehensive harm reduction programs substantially decrease HIV incidence among IDUs [14]. Countries such as the Netherlands, Sweden, Germany, France, Belgium, Denmark, and Switzerland report reductions of 15–35% in new IDU-related HIV cases over the past five years, with decreases exceeding 30% in the Netherlands and Sweden [4,14,15]. Conversely, limited access to harm reduction services in parts of Eastern Europe and Central Asia (EECA) has contributed to the fastest growing HIV epidemic worldwide, with new infections increasing by 48% between 2011 and 2021 and treatment coverage reaching only 51% in 2022 [16,17,18].

Against this epidemiological backdrop, Romania has made notable strides in HIV case management. The number of individuals receiving ART continues to increase, indicating expansion of treatment programs and enhanced adherence. The modest rise in patients under clinical follow-up—paired with a relatively stable incidence—suggests improved effectiveness of retention-in-care strategies. These advancements reflect broader Western European trends in which ART availability and optimized care pathways have transformed HIV into a manageable chronic condition, enabling near-normal life expectancy for those receiving treatment [11,12]. Correspondingly, Romania has observed a decline in in AIDS-related mortality, with deaths decreasing from 185 in 2022 to 125 in 2023. This aligns with developments in Western Europe, where universal ART access and strong healthcare systems continue to reduce HIV associated mortality. In contrast, mortality remains elevated in parts of Central and Eastern Europe, particularly Ukraine and Belarus. Conflict-related disruptions in Ukraine between 2022 and 2024 have significantly impeded access to ART; with up to 40% of treatment centers damaged or closed, UNAIDS and WHO project a 20–30% increase in HIV-related mortality compared with pre-conflict levels.

Mother-to-child transmission represents another critical element of Romania’s HIV response. The observed increase in vertical transmission—from a low of 0.8% in 2023 to 1.98% in 2024—signals the need for strengthened health education, consistent prenatal screening, and systematic monitoring of pregnant women living with HIV [19]. Persistent barriers, including reduced emphasis on HIV awareness programs, rising migration patterns affecting access to prenatal services, and stigma that may deter care-seeking behavior, contribute to elevated MTCT risk [4,6,7,19]. Sweden provides a notable example of successful intervention, maintaining one of Europe’s lowest MTCT rates (≈1% or lower) through universal access to ART, comprehensive prenatal support, and early detection strategies [1,8]. At the opposite extreme, the ongoing conflict in Ukraine has contributed to vertical transmission rates estimated at 4–6%, largely due to disruptions in clinical services and ART availability [4,6,19].

Taken together, these findings underscore the necessity of a comprehensive and integrated public health approach to HIV prevention and care. Heterosexual transmission continues to drive the majority of new infections, demanding strengthened sexual health education, expanded screening in high-risk populations, and greater promotion of preventive measures. The rising proportion of new cases among MSM requires targeted interventions, including increased access to PrEP and culturally tailored awareness programs [2,3]. The demonstrated effectiveness of harm reduction services among IDUs further highlights the need for sustained investment in NSPs and OST as key components of prevention strategies [20]. Finally, the ongoing decline in HIV-related mortality attests to the impact of ART and improved healthcare access, emphasizing the importance of continued efforts to optimize treatment adherence and ensure equitable access across regions and population groups [21,22].

It is also important to assess Romania’s progress toward the UNAIDS 95–95–95 targets, by using available indicators. The number of HIV infected patients estimated for Romania by WHO for 2024 was as high as 20,000, the lowest being approximately 16,000, very closed to reported data for the same year of 18,768, supporting the idea that in our country the rate of diagnosed patients is at least 86%, the average European rate [23].

By the end of 2024, 15,713 individuals living with HIV were receiving antiretroviral therapy, indicating an ART coverage of approximately 84% among diagnosed cases. This level reflects substantial progress toward the second UNAIDS benchmark, although it remains below the target of 95%, at comparable level with European Region, were 86% from HIV diagnosed individuals are treated, as Teymur Noori, European Center for Disease Control expert, showed at the la European AIDS Conference in October 2025 in Paris [24,25].

The rate of viral suppression among treated Romanian patients was 86%, namely 9996 out of 11,644 persons with available viral load in 2024, meaning that only 74% out of 15,713 treated Romanian patients accessed HIV monitoring. The European Region average of suppressed viral replication is 70% [26]. Despite the gaps in HIV monitoring, improvements in ART uptake, retention in care, and the decline in mortality in 2023 indicate strengthening of the HIV care continuum in Romania. Conversely, the rise in mortality in 2024 and persistent transmission among key populations underscore the need for intensified and sustained action. Further research is required to identify structural and behavioral barriers affecting linkage to care. From a policy perspective, expanding routine and community-based testing, integrating HIV services more effectively within primary care, scaling up PrEP and harm-reduction interventions, and addressing stigma within healthcare and community settings will be critical for accelerating progress toward the 95–95–95 goals and ensuring long-term epidemic control [27].

This study has several limitations that should be considered when interpreting the findings. First, the analysis relied exclusively on national surveillance data, which, although comprehensive, may be affected by underreporting or delayed reporting, particularly among key populations with limited access to healthcare or heightened stigma. Second, the limited assessment viral suppression prevented accurate evaluation of the third 95 target and restricting inferences about treatment effectiveness beyond ART uptake. Third, the descriptive design precludes causal interpretations and does not allow adjustment for potential confounding factors such as socioeconomic disparities, migration patterns, or regional differences in healthcare access. Additionally, the use of aggregated, publicly available datasets prevented examination of individual-level clinical outcomes. Finally, comparisons with European Union and Eastern European data were constrained by variations in surveillance systems, testing policies, and reporting practices across countries. Despite these limitations, the study provides an important and updated overview of HIV epidemiology in Romania and offers valuable insights for guiding public health strategies.

## 5. Conclusions

The findings of this study indicate that Romania’s HIV epidemic between 2022 and 2024 is characterized by both epidemiological stability and notable shifts in transmission dynamics. Although the national incidence remains below the European average, the modest rise in newly reported cases and the evolving distribution of transmission routes reflect an increasingly complex epidemiological landscape. The substantial growth in cases among men who have sex with men, from 3.91% in 2007 to 32% in 2024, underscores the need for strengthened, population-specific prevention strategies and broader implementation of evidence-based interventions, including PrEP and targeted sexual-health education.

At the same time, improvements in treatment coverage and follow-up reflect progressive consolidation of the national HIV care continuum. The continued expansion of antiretroviral therapy, alongside rising numbers of individuals retained in care, demonstrates enhanced system performance and alignment with European trends toward long-term chronic disease management. Despite this progress, the increase in HIV-related mortality observed in 2024 highlights the ongoing need to optimize early diagnosis, treatment adherence, and equitable access to care across regions and vulnerable groups.

Mother-to-child transmission, although occurring at low levels, exhibited an upward trend in 2024, pointing to gaps in prenatal screening, linkage to care, and access to preventive services among at-risk pregnant women. Addressing these gaps through targeted education, universal prenatal HIV testing, and sustained monitoring will be essential for preventing future increases.

Although injection-related transmission represents a small proportion of cases, international and national experience demonstrates that harm-reduction services remain indispensable for ensuring long-term control. Maintaining and expanding access to needle exchange program and opioid substitution therapy is therefore crucial.

At the same time, Romania recorded significant steps toward 95-95-95 UNAIDS benchmark and has made substantive progress in HIV prevention, diagnosis, and treatment over the past decades. However, the persistence of behavioral, structural, and health-system barriers necessitates continuous adaptation of national strategies. A comprehensive and integrated approach—encompassing expanded prevention services, improved health education, robust surveillance, and sustained investment in treatment infrastructure—is required to mitigate ongoing transmission and advance toward the long-term public-health goal of reducing HIV as a major health threat.

## Figures and Tables

**Table 1:** General data on HIV and AIDS infection in Romania (updated on 30 December 2024).

Indicator	Number of Cases
Total HIV/AIDS cases (1985–2024)	28,793
Total AIDS cases (1985–2024)	18,881
Total HIV cases (1992–2024)	9912
People lost from records	1276
Total AIDS deaths (1985–2024)	8749
People living with HIV/AIDS (PLWHA)	18,768
New cases (1 January 2022–30 December 2024)	2227
Deaths (1 January 2022–30 December 2024)	553

**Table 2:** New HIV and AIDS cases reported in Romania between 2022 and 2024.

Year	Total Cases	HIV	AIDS
2022	757	425	332
2023	760	457	303
2024	810	475	335
Total	2227	1357	970

**Table 3:** Number of people living with HIV by age group in 2024.

Age Group (Years)	Number of People Living with HIV/AIDS in 2024	Percentage (%)
0–14	141	0.75%
15–19	130	0.70%
≥20	18,290	98.55%
Total	18,560	100%

**Table 4:** Trends of HIV transmission between 2022 and 2024.

Transmission Route	2022 (Number of Cases, %)	2023 (Number of Cases, %)	2024 (Number of Cases, %)
Vertical	8 (1.07%)	6 (0.84%)	16 (1.97%)
MSM	220 (29.06%)	214 (28.15%)	247 (30.49%)
IDUs	50 (6.6%)	63 (8.28%)	42 (5.19%)
Heterosexual	467 (61.7%)	464 (61.05%)	481 (59.38)
Unspecified	9 (1.2%)	7 (0.92%)	6 (0.74%)
Total cases	757	760	810

**Table 5:** HIV incidence in Europe and Romania (2023–2024).

Indicator	Europe 2023	Europe 2024	Romania 2023	Romania 2024
New HIV cases	112,883	115,000	540	475
Incidence (cases/100,000 ppl.)	3.4	3.5	2.85	2.51
Eastern Europe share (%)	69%	70%	-	-
HIV/AIDS mortality (deaths)	15,420	14,890	125	193

**Table 6:** HIV and AIDS statistics for Romania between 2023–2024.

Indicator	2023	2024	Δ (%)
AIDS deaths	125	193	+54.4%
Patients followed up	15.265	18.768	+22.94%
Patients on ART	15.176	16.464	+8.49%

## Data Availability

The data presented in this study are publicly available in the report Evoluția HIV în România—31 December 2022, published by the National Commission for the Surveillance, Control and Prevention of HIV/AIDS (CNLAS), Romania. The report can be accessed online at: https://www.cnlas.ro/images/doc/31122022_rom.pdf (accessed on 26 January 2025).

## Abbreviations

The following abbreviations are used in this manuscript:

HIV	Human Immunodeficiency Virus
AIDS	Acquired Immunodeficiency Syndrome
ECDC	European Centre for Disease Prevention and Control
EU	European Union
MSM	men who have sex with men
IDU	intravenous drug user
ART	antiretroviral therapy
WHO	World Health Organisation
CDC	Center for Disease Prevention and Control
PLWHA	people living with HIV/AIDS
PrEP	pre-exposure prophylaxis
MTCT	mother-to-child transmission
OST	opioid substitution therapy
NEPs	needle exchange programs
EECA	Eastern Europe and Central Asia
